# The amnesic effects of propofol on functional connectivity in the hippocampus determined by functional magnetic resonance imaging in volunteers

**DOI:** 10.1016/j.bja.2025.04.032

**Published:** 2025-06-03

**Authors:** David Lindsay, Ram M. Adapa, David K. Menon, Emmanuel A. Stamatakis

**Affiliations:** 1 Division of Anaesthesia, University of Cambridge, Cambridge, UK; 2 Department of Clinical Neurosciences, University of Cambridge, Cambridge, UK; 3 Cambridge University Hospitals NHS Foundation Trust, Cambridge, UK; 4 Department of Medicine, University of Cambridge, Cambridge, UK

**Keywords:** amnesia, fMRI, general anaesthesia, hippocampus, memory, propofol, sedation

## Abstract

**Background:**

One of the primary actions of general anaesthetic agents, apart from inducing a state of unconsciousness, is reversible impairment of memory formation during the period of administration. Failure to induce and maintain amnesia can result in recall of accidental intraoperative awareness and contribute to adverse psychological health outcomes. The precise mechanisms of action by which general anaesthetics achieve their amnesic effects are not fully understood. To this end, we focused on the hippocampus, a region critical for the formation of new episodic explicit long-term memories of the type normally inhibited by general anaesthetics.

**Methods:**

We enrolled 25 healthy adult volunteers who underwent functional magnetic resonance neuroimaging (fMRI) whilst sedated with a plasma target-controlled infusion of the anaesthetic agent propofol. The functional connectivity (synchronised neuronal activity with other brain regions) of the hippocampus and microanatomical hippocampal subregions was assessed at baseline, under sedation, and during recovery. Serial plasma propofol concentrations and responses to an auditory stimulus semantic decision task were measured. Post-scanning memory testing was conducted, and memory performance was related to the fMRI data.

**Results:**

Functional connectivity changes associated with an amnesic but subhypnotic depth of propofol sedation were predominantly characterised by a reduced connectivity signature of the hippocampus stratum radiatum, stratum lacunosum, stratum moleculare, CA1 stratum pyramidalis, and CA4/dentate gyrus subfields with the precuneus.

**Conclusions:**

We provide evidence for differential actions of propofol on hippocampal subdivisions and limbic circuits related to amnesic efficacy, which suggests a more significant role of the precuneus in long-term memory consolidation than previously thought.

**Editor's key points**

• The mechanisms by which general anaesthetics achieve their amnesic effects are not fully understood, but actions on the hippocampus, a region critical for the formation of new memories, are thought to be involved.
• Healthy adult volunteers underwent functional magnetic resonance neuroimaging (fMRI) during target-controlled infusion of propofol, with analysis of functional connectivity at baseline, under sedation, and during recovery, with plasma propofol concentrations and responses to an auditory stimulus and post-scanning memory testing.
• Subregion-specific reduced hippocampal functional connectivity was associated with an amnesic but subhypnotic effect, with evidence for a more prominent role of the precuneus in explicit memory consolidation than previously suggested.

Since the advent of modern anaesthesia, the primary function of general anaesthetic agents has been to induce a rapid and fully reversible state of unconsciousness. However, one of the other major effects of general anaesthetics is anterograde amnesia, specifically inhibition of episodic explicit long-term memory formation, which can occur at subhypnotic dosages.^1^^,^^2^ Despite extensive clinical use, the mechanisms by which general anaesthetic drugs disrupt memory formation remain poorly understood.

Accidental awareness under general anaesthesia (AAGA) with recall remains an uncommon but potentially life-changing complication of general anaesthesia.^3^ For AAGA with explicit recall to occur, both failure of hypnosis and failure to prevent memory formation are required.

Just as the risk of AAGA varies between anaesthetic agents (albeit dwarfed by a range of confounding factors), the amnesic efficacy of anaesthetic drugs relative to their hypnotic potency also varies by agent. Previous experiments by Veselis and colleagues^1^^,^^2^^,^^4^ found both propofol and midazolam to be profoundly amnesic at subhypnotic doses, whereas thiopental and dexmedetomidine both had only mild amnesic effects, and fentanyl alone had effectively negligible memory effects. This is consistent with the hypothesis that thiopental and dexmedetomidine primarily cause amnesia by impairing perception and memory encoding through sedation and unconsciousness, whereas propofol and midazolam primarily act on early memory consolidation (the subsequent stage of the memory formation and storage sequence) to exert their amnesic effects, potentially independently of the level of sedation.^2^^,^^5^^,^^6^

A range of clinical case studies, animal experiments, and neuroimaging evidence have highlighted the central role of the hippocampus in early memory consolidation.^7^, ^8^, ^9^, ^10^ The hippocampi are bilateral, convoluted archicortical structures located in the medial temporal lobes of the brain.^11^ Localised lesions in this area acquired in adulthood are associated with catastrophic impairment of new episodic memory formation capacity, despite apparently normal short-term memory and recall of events from long before the lesion event.^9^

The hippocampi are histologically divided into several subregions running longitudinally.^11^, ^12^, ^13^ Different subdivisions have been associated with different memory functions in animal models at multiple scales, with a range of long-term spatial, pattern, and social memory types localised to specific mesoscopic subregions and at a cellular level, encoding engrams and spatial positioning information.^9^^,^^14^ Two major neural circuits have been identified within the hippocampus running from the entorhinal cortex to the subiculum: the monosynaptic direct pathway and the trisynaptic pathway. In addition, a third pathway has been described via the smaller CA2 region (Fig. 1).^9^ The direct pathway is thought to be sufficient for some types of memory formation without input from the trisynaptic pathway, which might facilitate rapid learning from single episodic experiences.^15^

**Fig 1:**
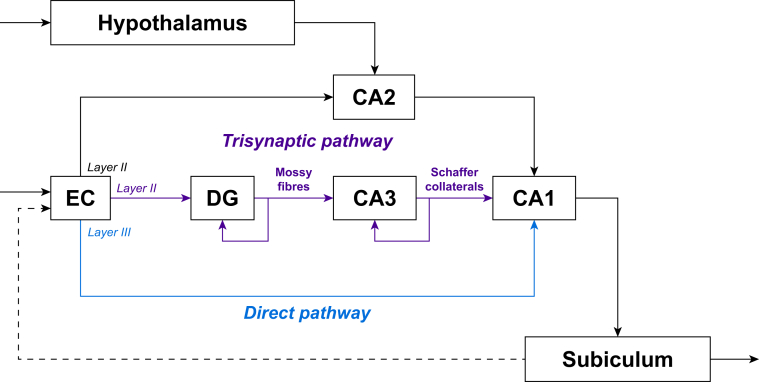
Schematic of simplified hippocampal formation internal circuits (major inputs and connections only; more minor pathways not shown). The direct pathway is highlighted in blue, and the trisynaptic pathway is depicted in purple. CA, cornu ammonis; DG, dentate gyrus; EC, entorhinal cortex.

Functional magnetic resonance imaging (fMRI) is a neuroimaging technique that utilises the difference in paramagnetic and diamagnetic susceptibility between deoxyhaemoglobin and oxyhaemoglobin, respectively, to infer changes in regional cerebral blood flow and, in turn, underlying changes in local neuronal activity.^16^^,^^17^ Pryor and colleagues^18^ have identified differential effects of propofol on anterior and posterior divisions of the hippocampus using blood oxygen level-dependent (BOLD) fMRI. Their findings include a decrease in posterior hippocampus activation proportionate to decreased memory performance.^18^

We hypothesise that propofol inhibits early memory consolidation by differentially acting on specific pathways within the hippocampus as a mechanism of propofol-induced amnesia. To characterise this, an experiment was performed using a novel microanatomical hippocampal subregion segmentation in a seed-to-voxel resting-state functional connectivity (FC) analysis. This allowed us to investigate the effects of propofol sedation on hippocampal subregion functional connectivity in association with quantified drug-induced amnesic effects and measured plasma propofol levels. The results offer insights into both the mechanisms of general anaesthetic action and why those mechanisms sometimes fail.

## Methods

The experimental protocol used for primary data acquisition has been described previously^19^^,^^20^ and is summarised below.

Twenty-five healthy, right-handed, native English-speaking, adult volunteers were recruited and assessed before written informed consent for study participation was obtained (age range 19–53 yr, mean 34 yr; sex ratio 11:14 male:female). Ethical approval for this study was prospectively confirmed by the Cambridgeshire 2 Research Ethics Committee. All volunteers were required to follow standard fasting guidelines and safety procedures. Two senior anaesthetists and dedicated monitoring equipment provided continuous monitoring throughout. The performance, safety, and magnetic resonance (MR) image quality profiles of the target-controlled infusion (TCI) pharmacokinetic pumps (Carefusion, Alaris Products, Basingstoke, UK) were evaluated inside the MR scanner suite and confirmed to be within accepted limits in an earlier study.^21^ As the study was originally powered for a different primary analysis, sample size was not specifically predetermined for the work reported here.^20^

### Sedation

Volunteers were sedated with propofol (Fresenius Propoven 1%, Fresenius Kabi, Cheshire, UK) using a Marsh-model propofol TCI to a sedation target of CpT = 1.2 μg ml^−1^, with a period of 10 min included to allow for plasma-effect site equilibration.^22^ Plasma propofol concentrations were measured on blood samples drawn after the equilibration period for the sedation phase and after recovery-phase imaging (initiated 20 min after propofol infusion was terminated) using high-performance liquid chromatography.^19^ Pharmacokinetic model accuracy was assessed using mean prediction error (equation (1)) and median absolute performance error (equation (2)) based on measured (mCp) and model-predicted (Cp) plasma propofol concentrations.^22^^,^^23^

(1) $${PE}_{PK}=\frac{mCp–Cp\;}{Cp}\times 100$$

(2) $${APE}_{PK}=\left|{PE}_{PK}\right|$$

### Memory and behavioural testing

At the baseline and sedation stages, volunteers were presented with auditory word stimuli using the Cognition and Brain Sciences Unit Audio Stimulation Tool, and they were required to select whether the word referred to a ‘living’ or ‘non-living’ object using a button-box immediately before acquisition of resting-state sequences.^19^ ‘Time-out's’ (no response within 3 s), error rate, and response time were recorded in real time. Memory testing was performed when volunteers were fully recovered and a minimum of 30 min after cessation of propofol infusion using a mixture of old (previously heard) and new word stimuli. The Explicit Recall Sensitivity Index (ERSI), dʹ (the difference between the z-transformed ‘hit rate’, H, which is the proportion of times a subject correctly identified a stimulus as previously heard, and the ‘false alarm rate’, F, which is the proportion of times a subject incorrectly identified a stimulus as old when it was actually new), was calculated using equation (3).^18^

(3) d'=z(H)–z(F)

Memory and behavioural data were related to plasma propofol target concentration using Cohen's d and Wilcoxon signed-rank tests and to measured plasma propofol concentration using Spearman's rank correlation coefficient, linear regression, and Cohen's f^2^ statistical tests.

### Image acquisition

Structural and resting-state BOLD functional MR images were acquired using a Siemens Trio Tim 3 Tesla MRI System (Wolfson Brain Imaging Centre, Cambridge, UK). Each functional run (baseline, sedation, and recovery phases) included 150 volumes of 32 interleaved 3 mm slices, with an interslice gap of 0.75 mm, a resolution of 3 mm × 3 mm × 3 mm, an acquisition time of 2 s, an echo time of 30 ms, a flip angle of 78°, and a bandwidth of 2442 Hz Px^-1^. T1-weighted structural images were acquired using a magnetisation-prepared rapid gradient echo (MPRAGE) sequence with a resolution of 1 mm × 1 mm × 1 mm, a repetition time of 2250 ms, an inversion time of 900 ms, an echo time of 2.99 ms, and a flip angle of 9°.

### Image processing and analysis

After removing the first five volumes of each functional run, MR images were preprocessed using SPM12 (Statistical Parametric Mapping v12, update revision 7771, Wellcome Centre for Human Neuroimaging, UCL, London, UK) running on MATLAB R2021b (9.11.0.1769968) (MathWorks, Natick, MA, USA). The slice-timing corrected, realigned, and spatially normalised functional images were written to a super-sampled final resolution of 2 mm × 2 mm × 2 mm.

Functional connectivity analysis was performed using regressor time series extracted from unsmoothed images using the CONN Functional Connectivity Toolbox (version CONN22.a) and its default FSL Harvard–Oxford Atlas and Automated Anatomical Labelling atlases.^24^^,^^25^ The McGill Computational Brain Anatomy Laboratory (CoBrALab) 3T *in vivo* High Resolution Atlas of the Hippocampus and Subfields was used to generate hippocampus subfield regions of interest (ROIs).^26^, ^27^, ^28^ Because of MRI atlas resolution limits, several histological subfields are grouped into joint ROIs: CA2 and CA3 stratum pyramidalis subfields (CA2/CA3), CA4 stratum pyramidalis and dentate gyrus subfields (CA4/DG), and stratum radiatum/stratum lacunosum/stratum moleculare layers (Stratum). Other mapped ROIs include the CA1 stratum pyramidalis subfield, subiculum, alveus, fimbria, fornix, mammillary bodies, and the amygdala. Denoising (aCompCor), motion regression, BOLD time series band-pass frequency filtering (0.008–0.09 Hz), and linear detrending pipelines were applied as implemented in CONN.

Within-subject seed-to-voxel connectivity analysis was performed using weighted general linear models (GLMs) for each seed-target area by computing the correlations between each ROI's time series and brain-wide target voxels. Between-subject group analysis was performed using GLMs estimated for each voxel with relevant first-level connectivity measures for each subject.

Voxel cluster-level inferences were performed using Gaussian random field theory two-sided parametric statistics.^29^ Unless stated otherwise, results are reported with statistical thresholding set at *P*<0.001 uncorrected cluster-forming voxel-level threshold and *P*-FDR <0.05 cluster-size threshold, corrected for multiple comparisons using false discovery rate correction.^17^^,^^30^

## Results

**Fig 2:**
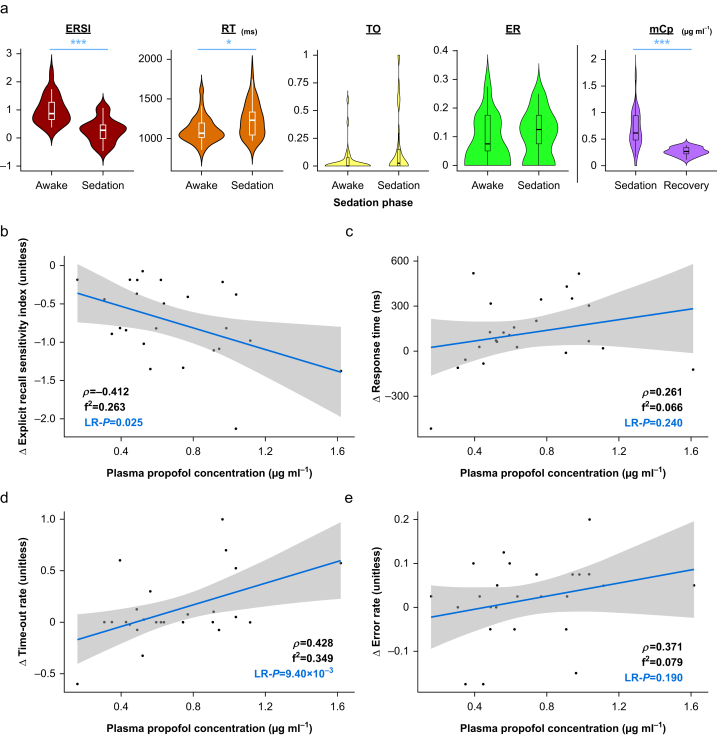
(a) Violin box plots of the ERSI, RT, TO, ER, and measured plasma propofol concentration (mCp) by experimental phase (∗*P*<0.05, ∗∗*P*<0.01, ∗∗∗*P*<0.001). Change from awake baseline in (b) ERSI, (c) RT, (d) TO, and (e) ER against measured plasma propofol concentration during pseudo-steady-state TCI sedation with linear regression (blue) and 95% confidence intervals (grey). ER, error rate; ESRI, Explicit Recall Sensitivity Index; f^2^, Cohen's f^2^; LR-*P*, linear regression *P*-value; RT, response time; target-controlled infusion; TO, time-out rate; *ρ*, Spearman's rank correlation coefficient.

The mean (sd) measured plasma propofol concentration (mCp) across all participants was 0.70 (0.32) μg ml^−1^ during sedation (CpT=1.2 μg ml^−1^) and 0.27 (0.077) μg ml^−1^ during the recovery phase (Wilcoxon signed-rank test *P*=4.2×10^−4^) (Fig. 2a). The mean prediction error (a measure of bias) for the pharmacokinetic model was –42%. The median absolute performance error (a measure of precision) was 52%.

In all cases, the memory performance of the sedation period stimuli was decreased compared with pre-sedation baseline, with a mean (sd) decrease in Explicit Recall Sensitivity Index from 1.00 (0.488) to 0.264 (0.413) (*P*=1.9×10^−5^) (Fig. 2a). Mean (sd) response time increased from 1110 (160) ms pre-sedation to 1230 (206) ms for sedation (*P*=0.011). During propofol infusion, several volunteers exhibited markedly decreased responsiveness to auditory stimuli, with a time-out rate of 100% in one individual. However, most remained fully responsive with a modal time-out rate of 0% and a median time-out rate of 2.5% (*P*=0.098). The difference in error rate was also not statistically significant (*P*=0.18).

Propofol infusion (CpT) had a proportionately larger effect on explicit memory performance (Cohen's d=1.60) than any proxy marker of responsiveness or cognitive performance, at 2.5 times greater than the effect on response time (d=0.65), 3.0 times greater than the effect on time-out rate (d=0.53), and 6.8 times greater than the effect on error rate (d=0.23) for the semantic decision task at a group level. Changes in memory performance and responsiveness also correlated with measured plasma propofol concentration (mCp) in all domains, although this was not statistically significant in the case of response time and error rate (Fig. 2b–e). A previous analysis demonstrated no detectable priming or familiarity implicit memory component in memory testing responses.^31^

Resting-state fMRI (rs-fMRI) responses have been shown to correlate with task performance.^32^^,^^33^ No significant functional connectivity patterns were identified as attributable to confounding factors when performing a regression analysis for age, sex, height, or weight in addition to motion regression in functional images.

**Fig 3:**
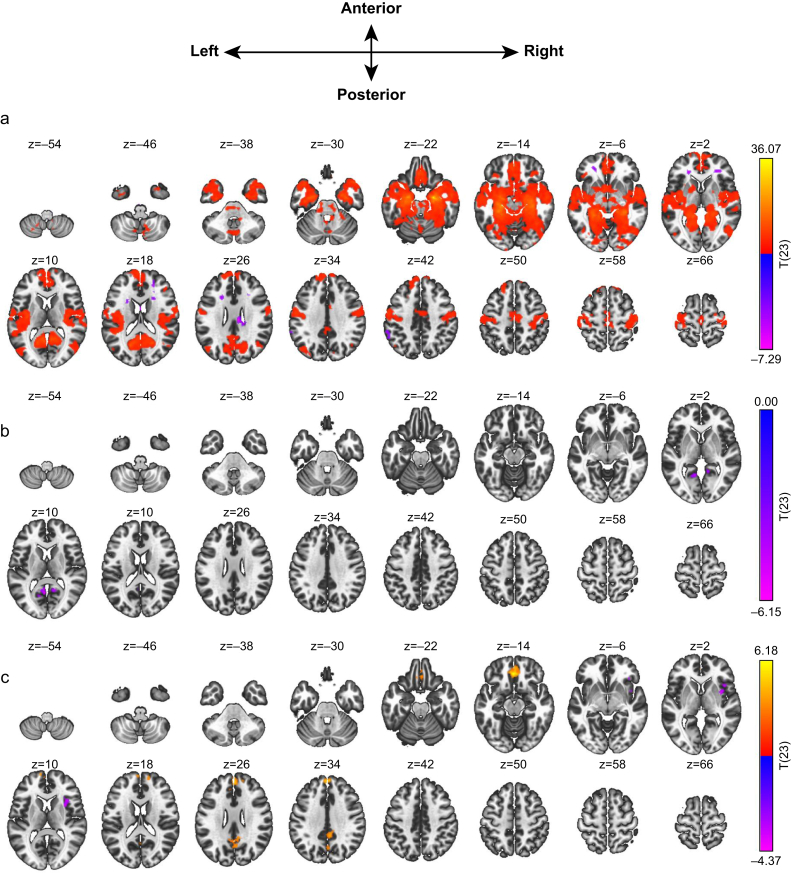
Whole hippocampus ROI, with resting-state functional connectivity overlay on a standard structural image (colour scale key: T-statistic; slice levels: Montreal Neurological Institute z-axis coordinate): (a) baseline, (b) difference of sedation > baseline, and (c) difference of recovery > sedation (voxel threshold *P*-uncorrected <0.01 in c). ROI, region of interest.

As one of the most critical structures required for new episodic long-term memory formation, resting-state functional connectivity of the whole hippocampus was assessed at baseline (pre-sedation) using a left and right averaged ROI time series generated using the Harvard–Oxford atlas. The hippocampus as a whole displayed a positive activity correlation (i.e. connectivity) with the precuneus, right precentral gyrus, brainstem, left frontal orbital cortex, and right frontal pole (Table 1 and Fig. 3a). Assessing the effect of propofol sedation on overall hippocampus functional connectivity, a paired *t*-test analysis between the sedation and baseline conditions demonstrated a significant decrease in connectivity with two clusters in the precuneus (Table 1 and Fig. 3b).

**Table 1:** Whole hippocampus ROI, resting-state functional connectivity clusters with correlation at (a) baseline and with change in functional connectivity comparing (b) difference of sedation > baseline and (c) difference of recovery > sedation (voxel threshold *P*-uncorrected <0.01 in c [above generally accepted significance thresholds]). FC, functional connectivity; FDR, false discovery rate; MNI, Montreal Neurological Institute; ROI, region of interest.

Cluster rank	MNI coordinates			Cluster size (voxels)	Size *P*-FDR	Correlation (+/–) / FC change (↑/↓)	Main location
	x	y	z				
Whole hippocampus baseline FC							
1	+22	–08	–22	47069	<1×10^−6^	Positive	Precuneus
2	–04	–06	+54	1185	<1×10^−6^	Positive	Right precentral gyrus
3	–04	–16	–46	345	<1×10^−6^	Negative	Brainstem
4	–30	+30	–12	171	7.7×10^−5^	Positive	Left frontal orbital cortex
5	+26	+16	+16	143	2.5×10^−4^	Negative	Right frontal pole
6	–54	–36	+38	137	2.8×10^−4^	Negative	Left posterior supramarginal gyrus
7	+08	–20	+26	132	3.1×10^−4^	Negative	Posterior cingulate gyrus
8	+30	+32	–14	124	4.2×10^−4^	Positive	Right frontal pole
9	–20	+16	+22	112	7.2×10^−4^	Negative	White matter (left frontal lobe)
10	–22	+36	+02	91	2.2×10^−3^	Negative	White matter (left frontal lobe)
11	–28	–68	–62	82	3.4×10^−3^	Positive	Left cerebellum
12	00	–02	+18	73	5.5×10^−3^	Negative	Corpus callosum
13	+42	+32	+16	57	1.5×10^−2^	Positive	Right inferior frontal gyrus
14	+30	+36	+04	54	1.7×10^−2^	Negative	White matter (right frontal lobe)
Whole hippocampus difference of sedation > baseline							
1	–04	–58	+10	241	2.9×10^−5^	↓	Precuneus
2	+10	–52	+08	76	4.1×10^−2^	↓	Precuneus
Whole hippocampus difference of recovery > sedation							
1	+02	+54	+30	465	4.9×10^−4^	↑	Left frontal pole
2	+06	–44	+32	454	4.9×10^−4^	↑	Posterior cingulate gyrus/precuneus
3	–02	+36	–14	359	1.9×10^−3^	↑	Frontal medial cortex
4	+46	+08	–02	346	1.9×10^−3^	↓	Right insular cortex

The effect of stopping propofol sedation was also assessed for potential reciprocal changes. A paired *t*-test between recovery and sedation conditions demonstrated an increase in functional connectivity with the fronto-medial cortex and right superior frontal gyrus. Anticipating a return of functional connectivity with the precuneus, relaxing the voxel threshold to *P*-uncorrected <0.01 displayed reciprocally increased connectivity with the posterior cingulate gyrus and precuneus, albeit more superiorly than the focus of decreased functional connectivity observed during sedation (Table 1 and Fig. 3c). However, this threshold does not meet generally accepted significance levels. A paired *t*-test between the recovery and baseline conditions did not show any significant functional connectivity changes from baseline, suggesting an effective return to normal rather than subnormal or supranormal functional connectivity.

To investigate the role of individual hippocampal subregions, the hippocampus was divided into separate subfield ROIs using the CoBrALab 3T *in vivo* High Resolution Atlas of the Hippocampus and Subfields.^26^, ^27^, ^28^ Three subfields were identified, which corroborated the pattern of decreased functional connectivity with a cluster centred on the precuneus during sedation, as observed in the whole hippocampus ROI: CA1, the subiculum, and the combined ‘Stratum’ subregions (Table 2). The CA4/dentate gyrus subfield also displayed decreased functional connectivity with the precuneus, but this did not reach robust statistical significance. The subiculum demonstrated a rebound increase in functional connectivity with the precuneus and posterior cingulate gyrus when contrasting the recovery and sedation phases. However, this was only at a relaxed voxel threshold (*P*-uncorrected <0.005), whereas the CA1 and Stratum subfields did not show any reciprocal functional connectivity change during recovery.

**Table 2:** CA1, subiculum, and ‘Stratum’ hippocampus ROIs, resting-state functional connectivity clusters, difference of sedation > baseline; whole hippocampus, ‘Stratum’, CA4/dentate gyrus, and CA1 subfield hippocampus ROIs, resting-state functional connectivity clusters, difference of sedation > baseline with Explicit Recall Sensitivity Index dʹ beta-coefficients (voxel threshold *P*-uncorrected <0.005 in CA4/DG and <0.01 in CA1). FC, functional connectivity; FDR, false discovery rate; MNI, Montreal Neurological Institute; ROI, region of interest.

Cluster rank	MNI coordinates			Cluster size (voxels)	Size *P*-FDR	FC change	Main location
	x	y	z				
Stratum subfield difference of sedation > baseline							
1	–06	–50	+10	142	2.5×10^−3^	↓	Precuneus
CA1 subfield difference of sedation > baseline							
1	–06	–50	+10	118	9.4×10^−3^	↓	Precuneus
Subiculum subfield difference of sedation > baseline							
1	–06	–58	+14	122	5.6×10^−3^	↓	Precuneus
Whole hippocampus difference of sedation > baseline factoring ERSI (dʹ)							
1	–10	–16	+18	118	2.6×10^−3^	↑	Left Thalamus
Stratum subfield difference of sedation > baseline factoring ERSI (dʹ)							
1	+02	–68	+46	202	9.6×10^−5^	↓	Precuneus
2	–10	–16	+20	103	5.4×10^−3^	↑	Left thalamus
CA4/DG subfield difference of sedation > baseline factoring ERSI (dʹ)							
1	+12	–64	+38	311	5.5×10^−4^	↓	Precuneus
CA1 subfield difference of sedation > baseline factoring ERSI (dʹ)							
1	–18	–58	+24	628	7.6×10^−5^	↓	Precuneus
2	–40	–20	+26	300	1.2×10^−2^	↑	Left postcentral gyrus
3	–10	–16	+20	258	1.8×10^−2^	↑	Left thalamus

To separate the BOLD functional connectivity changes specific to the reduced memory function observed from other more general sedation-related and nonspecific neurovascular effects, the volunteers' Explicit Recall Sensitivity Index (dʹ) scores, which can be considered a proxy measure of accurate explicit memory formation, retention, and recall, were also incorporated into the GLM. Two further volunteers for whom a change in dʹ score or response time could not be calculated (time-out rate >75%) were excluded from further analysis.

**Fig 4:**
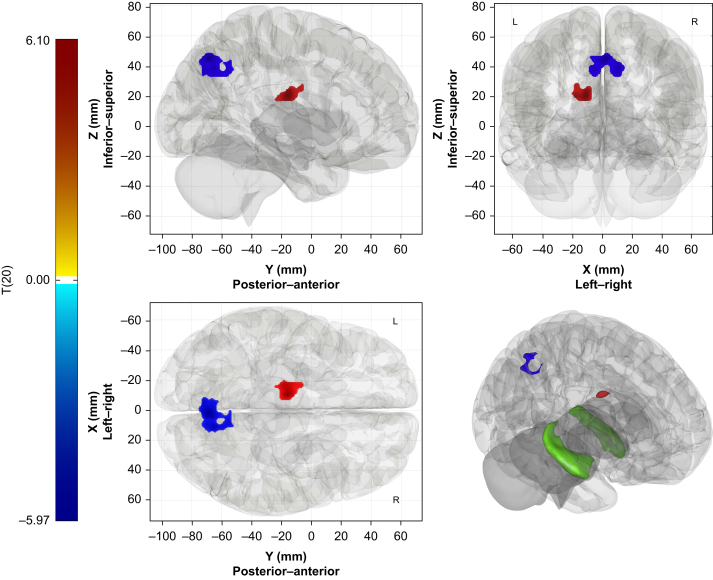
Change from baseline in functional connectivity of the combined ‘Stratum’ ROI under sedation weighted by change in Explicit Recall Sensitivity Index. Orthogonal views of statistically significant clusters with 3D reconstruction, including the whole hippocampus (green) (colour scale key: T-statistic; coordinates: Montreal Neurological Institute). ROI, region of interest.

When weighting for the dʹ scores, the only functional connectivity change during sedation observed for the hippocampus as a whole was an increase in connectivity with a cluster centred in the thalamus. However, some hippocampal subfields continued to display a decrease in functional connectivity with the precuneus. The combined Stratum subfield displayed decreased functional connectivity with a cluster in the superior aspect of the precuneus and increased connectivity with the thalamus (Table 2 and Fig. 4). The CA4/dentate gyrus and CA1 subfields displayed similar functional connectivity changes but were statistically subthreshold. The subiculum did not show any statistically significant functional connectivity changes.

To further separate amnesia-specific functional connectivity changes from general sedation and nonspecific changes, the change in response time, which can be considered a proxy measure of alertness and cognitive processing speed, and measured plasma propofol concentrations (mCp) were also included in the GLM in turn. There was no statistically significant change in functional connectivity attributable to alertness (response time) *per se*. mCp-related changes included an increase in functional connectivity between the whole hippocampus and the medial prefrontal cortex, right inferior temporal gyrus, and cerebellum during sedation and a decrease in functional connectivity with the right postcentral gyrus on recovery from sedation, which was also reflected in the main hippocampal subfields individually.

## Discussion

There are two main findings from this analysis of hippocampal functional connectivity during propofol sedation in humans. Firstly, propofol sedation-induced amnesia was predominantly associated with reduced functional connectivity of the stratum radiatum/lacunosum/moleculare, CA1 field stratum pyramidalis, and CA4 stratum pyramidalis/dentate gyrus subfields. Secondly, propofol sedation-induced amnesia was associated with reduced functional connectivity between the hippocampus and the precuneus.

Within the hippocampus, two major neuronal pathways are recognised: the direct pathway and the trisynaptic pathway.^9^ They both originate in the entorhinal cortex and converge on a common outflow tract to the subiculum from the CA1 pyramidal subfield. The direct pathway neurones synapse directly in the CA1 stratum pyramidalis field, whereas the trisynaptic pathway first synapses in the dentate gyrus and incorporates the mossy fibres connecting the dentate gyrus to the CA3 region and the Schaffer collaterals connecting the CA3 and CA1 regions (Fig. 1).^11^ The physiological roles of each of these pathways, and how interdependent they are, remain to be elucidated.

Although propofol might be acting on one or both of the direct and trisynaptic pathways, given the relative lack of functional connectivity changes observed in the CA2/CA3 regions, the changes in functional connectivity that we observed only support the former. This action might either be on the main circuit neurones themselves or be through effects on the interneurones, which are thought to make up around 10% of the cells in the CA1 region.^11^

The observed pattern of prominent functional connectivity changes in the CA1 and CA4/dentate gyrus regions is not unexpected as this would be consistent with the known distribution of gamma-aminobutyric acid type A (GABA_A_) receptors (and *N*-methyl-D-aspartate [NMDA] glutamate receptors), which are expressed at significantly greater densities within the CA1 and dentate gyrus subregions relative to other hippocampal areas.^11^ GABAergic interneurones are thought to be both a specific target of propofol and critical for hippocampal memory function.^34^^,^^35^

The CA1 region is a common nexus of both the direct and trisynaptic pathways, and it is known from previous clinical investigations in humans with lesions localised to the CA1 field (which is thought to be particularly susceptible to hypoxic, metabolic, and excitotoxic insults) that damage to this area results in severe deficits in episodic explicit long-term memory.^7^ This provides further evidence for a specific focal action of propofol, rather than nonspecific actions, in which case longer, polysynaptic pathways would logically be more vulnerable to disruption.

The precuneus is increasingly considered to be a major centre involved in memory.^36^^,^^37^ It is closely associated with the posterior cingulate cortex and the rest of the Default Mode Network (DMN), which is thought to have a role in autobiographical memory more generally.^8^^,^^33^

Reduced functional connectivity between the CA1 field stratum pyramidalis, stratum radiatum, stratum lacunosum, stratum moleculare, CA4 and dentate gyrus subregions, and the precuneus appears to be a characteristic feature of propofol-induced amnesia at subanaesthetic plasma concentrations.

Given that previous studies have associated propofol sedation with impairment of early memory consolidation specifically, these results support a role for the precuneus in normal memory consolidation function.^4^, ^5^, ^6^ The absence of significant functional connectivity changes between hippocampal subregions and auditory association areas might also be interpreted as a lack of evidence for disruption of initial engram encoding, further supporting an action of propofol on the consolidation phase specifically as the basis for impaired recall.

The precuneus could potentially serve a computational or integrative association role in memory consolidation or any downstream events directly. Alternatively, it might have a simpler role as a passive relay or distribution hub for long-range connections between the hippocampi and memory-associated regions, such as the prefrontal cortex, or with longer-term memory storage sites in the neocortex and source areas for the brain states being encoded and consolidated in memory. By implication, propofol might exert its amnesic action either by disrupting neuronal signalling between these areas or by acting at one or more of these sites directly. This would also be consistent with previous research suggesting that EEG changes observed to correlate with the amnesic effects of propofol, such as in the P2–N2 event-related potential and θ-synchrony, reflect underlying memory-related hippocampal–cortical feedback loops.^5^ As suggested by previous studies, this amnesic action might be independent of its sedative action.^1^^,^^2^^,^^4^^,^^5^^,^^18^

The hippocampus has been shown to have a large degree of structural and functional connectivity with thalamic nuclei associated with the extended hippocampal memory system, especially the anterior nuclear group and the mediodorsal nucleus.^38^ If indeed specific to memory changes, the observed increase in functional connectivity between the hippocampus and this region during sedation could reflect changes in upstream or downstream memory regulation or feedback resulting from subcircuit isolation. The connectivity changes observed when weighting for mCp might represent more general resting-state network changes. Taken together, this could indicate a division of the DMN, with fractional decoupling of the memory-specific components attributed to propofol.

All experimental results should be interpreted with caution. These results are from a single dataset, so they should be reproduced independently with other subjects and other sedative agents before firm conclusions can be drawn. As with most fMRI data, this dataset featured some artifacts, such as warping related to single-direction phase encoding and aliasing artifacts (wrapping) in some cases. Even after correcting for these, this analysis is pushing the limits of 3T BOLD fMRI. Unlike the neocortex, the hippocampus is mostly only trilaminar. It is also one of the most highly convoluted areas of the brain. With an isotropic resolution of 3 mm in the raw fMRI images and a 0.75 mm gap between slices in the z-axis, some partial volume effects and oversmoothing are inevitable for very fine anatomical structures, such as hippocampal subfields. In particular, the CA1 field stratum pyramidalis, stratum radiatum, stratum lacunosum, stratum moleculare, and CA4 and dentate gyrus subregions are all anatomically colocated in 3D Euclidean space (together with other histologically separate layers such as the stratum oriens) and typically have common or closely related vascular supplies.^9^^,^^11^, ^12^, ^13^^,^^39^ The differential underlying GABA receptor density in each ROI might also exert an influence. Whether these findings are truly specific effects on memory circuits or predominantly a consequence of the high baseline resting-state functional connectivity between the hippocampi and the precuneus (which also has a high resting regional cerebral blood flow) remains unclear.^40^ Given the increase in functional connectivity observed with the medial prefrontal cortex during sedation, when measured plasma propofol levels were included in the GLM, effects on the DMN more generally cannot be excluded.

Although explicit recall testing was only performed after volunteers had fully recovered clinically (a minimum of 30 min after the propofol was stopped), as this was tested during the same session, persisting subclinical drug effects of residual circulating propofol are possible. However, previous research suggests that this is unlikely to be significant.^5^ The mean mCp during infusion was notably 42% lower than predicted plasma propofol concentration (Cp), pharmacokinetically modelled at the time by the pump. The pharmacokinetic model appears to have performed less well in terms of both accuracy and precision than generally reported.^22^ This might be related to the target being lower than the dose range typically targeted for surgical planes of anaesthesia, as used in most pharmacokinetic studies, being at the lower extreme of the model envelope and amplifying the effects of fixed errors. Although the change in ERSI was statistically significant when compared against both CpT and mCp, the change in response time was significant between CpT targets but only weakly correlated with mCp and *vice versa* for time-out rate, an observation that also reflects pharmacokinetic model performance.

Some of the results observed, although physiologically plausible, fell short of statistical significance with the analytical methods used. However, it should be noted that as the hippocampal subfield ROIs are different from those ROIs originally anticipated during experimental design, this analysis could be underpowered. A task/event-related fMRI design alternative to resting-state fMRI would have allowed for the separation of remembered and forgotten words rather than an overall memory performance index. However, the focus on drug-induced changes in functional connectivity in this case and their relationship to the extended process of explicit memory formation makes resting state an advantageous approach.

Overall, this study provides a novel insight into the relative subregional actions of propofol on the human hippocampus *in vivo* at clinically relevant plasma concentrations sufficient to induce anterograde amnesia but below those required to completely inhibit response to auditory stimuli and decision-making performance. It also provides evidence for a more prominent role of the precuneus in explicit memory consolidation than previously suggested.

## Authors’ contributions

Experiment conception and design: DL, RA, DKM, EAS

Volunteer recruitment: RA, DKM, EAS

Data collection: RA, DKM, EAS

Data analysis: DL

Writing the manuscript: DL

Review of the draft manuscript: RA, DKM, EAS

Review of the final version of the manuscript: all authors

## Funding

The 10.13039/100010269Wellcome Trust (083660/Z/07/Z to RA); Raymond & Beverly Sackler Foundation Studentship; 10.13039/501100003342Cambridge Commonwealth Trust (to RA); The British Oxygen Professorship, The 10.13039/501100001297Royal College of Anaesthetists (to DKM); The Evelyn Trust (to DKM); The Canadian Institute for Advanced Research (CIFAR RCZB/072 RG93193 to DKM and EAS); Cambridge National Institute for Health and Care Research Biomedical Research Centre (to DKM); The Stephen Erskine Fellowship, Queens' College, University of Cambridge (to EAS).

## Declaration of interest

The authors declare that they have no conflict of interest.
